# Contribution and influence of social capital on corruption in the health sector: a view through the lens of service users

**DOI:** 10.1136/bmjgh-2025-020195

**Published:** 2025-12-09

**Authors:** Chinelo Esther Obi, Iheomimichineke Ojiakor, Chukwudi Nwokolo, Muktar Gadanya, Martin McKee, Blake Angell, Dina Balabanova, Obinna Onwujekwe

**Affiliations:** 1 Health Policy Research Group, University of Nigeria, Enugu, Nigeria; 2 Health Administration and Management, University of Nigeria, Enugu, Nigeria; 3 Centre for Entrepreneurship and Development Research, University of Nigeria, Nsukka, Nigeria; 4 Department of Community Medicine, Bayero University Kano, Kano, Nigeria; 5 Health Services Research and Policy, London School of Hygiene & Tropical Medicine, London, UK; 6 The George Institute for Global Health, Newtown, New South Wales, Australia; 7 Global Health and Development, LSHTM, London, UK

**Keywords:** Health services research, Health systems

## Abstract

**Background:**

The interplay between social capital and health system corruption in healthcare systems is underexplored, yet vital, a major barrier to advancing universal health coverage is underexplored. While social capital can support anticorruption by enabling collective action, it may also perpetuate corruption when networks are used to bypass procedures or access care unfairly. This study explores how social capital contributes to corrupt practices complex relationships play out in practice in Nigerian health facilities.

**Method:**

We conducted a cross-sectional study across two Nigerian states, surveying 1659 households using a pretested interviewer-administered questionnaire. A composite measure of social capital was developed from key variables. We examined the relationship between social capital and engagement in corrupt practices, controlling for variables such as gender, age, education and group memberships. Data were analysed using univariate, bivariate and multivariate methods.

**Results:**

Among respondents, 60.3% were female, over 90% had formal education, and 75.6% lived with their spouse. Nearly half (49.6%) had experienced corruption when accessing healthcare. Of those who used connections to receive care, 11.8% did so specifically to secure treatment. Individuals relying on family and friends (69.7%) were more likely to engage in corruption, and those using political connections always did. Non-membership in professional associations and political parties significantly reduced the likelihood of corrupt behaviour by 57% and 36%, respectively (p<0.01). Active participation in religious organisations was linked to lower corruption, while involvement in political parties, governance structures and professional bodies increased the likelihood. Higher education was associated with a 90% increased chance of engaging in corrupt practices (p<0.01).

**Conclusion:**

Social capital has both enabling and constraining effects on corruption in Nigeria’s healthcare system. Understanding its context-specific nature is crucial for designing effective anti-corruption strategies in health service delivery.

**WHAT IS ALREADY KNOWN ON THIS TOPIC**

- It is known that social capital engenders communal help and cohesion, which can potentially extend to the health system. However, there is paucity of information on how social capital enables corrupt practices in the health sector, supporting the need for new knowledge to be produced that will show how social capital can drive corruption in the health system.

**WHAT THIS STUDY ADDS**

- This study identifies the link between social capital and corruption in the health sector, especially the forms of social capital used by service users to access healthcare, including the categories of individuals more likely to engage in or facilitate corrupt practices.

**HOW THIS STUDY MIGHT AFFECT RESEARCH, PRACTICE OR POLICY**

- The findings offer valuable insights into the role of social capital in the health sector, providing guidance for future research and informing anticorruption strategies and policies that promote accountability without undermining community support systems.

## Introduction

 In many contexts, corruption in healthcare systems significantly hampers the provision and responsiveness of care. Corruption is defined as the abuse of power for private gain, encompassing both illicit and unethical practices that compromise equity, quality of care and public trust.^1 2^ For instance, in Nigeria, Agwu *et al*’s study in 2023 demonstrated how unaccountability and corrupt practices undermined frontline health workers’ ability to respond effectively during the pandemic, emphasising the need for systemic reforms.^3^ Similarly, studies from Montenegro^4^ and South Asia, including Bangladesh, India and Pakistan,^5^ reveal how pervasive corruption in healthcare delivery systems leads to diminished equity, poor-quality care and inadequate financial protection for patients. Corruption can manifest in various forms and understanding the drivers of these corrupt practices and how they operate across systems offers further insights into how corruption undermines health systems globally.^6 7^ Social capital refers to the valued connections, trust, shared values and networks within a society that facilitate social interactions and exchanges. Identified as a social determinant of health, social capital can impact health through pathways like social support, which provides emotional and material assistance and promotes healthier lifestyle behaviours such as diet, exercise and sleep.^8^,^11^

However, while social capital can promote good health and enhance trust in healthcare services, it can also foster corruption where personal connections and networks are leveraged to gain preferential treatment or bypass established processes in the healthcare system.^12 13^ This occurs particularly when social capital, instead of promoting collective action for the common good, becomes concentrated in certain groups with particular characteristics seeking to maximise the benefits for their members. This leads to inequalities as some groups can use their networks—kinship, social, political to gain privileged access to essential healthcare resources, services or information that would otherwise be difficult to obtain.^14 15^ In such cases, informal networks that typically foster trust can become mechanisms for exclusion and collusion for those without strong social connections.^14^ This excludes those without similar networks and aggravates existing power imbalances, hindering broader efforts to promote transparency and equity. Over time, using these personal relationships and networks to obtain preferential access to services creates systemic inequalities, reinforcing corruption and reducing equitable access to healthcare.^12^ Therefore, there are indications that the misuse of social capital may become a significant mechanism by which corruption is perpetuated within the health sector.

While some data have emerged, the influence of social capital on corruption within the healthcare system remains underexplored, particularly in the context of achieving Universal Health Coverage (UHC) as outlined in the Sustainable Development Goals. Corruption in the healthcare system undermines efforts to achieve equitable access to healthcare, a core objective of UHC, acting as a significant barrier to healthcare services.^16^ The relationship between social capital and corruption—and the pathways through which this relationship shapes access to and delivery of healthcare services—remains poorly understood.^17 18^ Such an understanding is vital not only to generate evidence on the nature of the problem but also to inform the development of interventions that can address drivers of corrupt behaviours and feasibly work to overcome them.

Empirical evidence from diverse studies sheds light on the complex relationships between social capital, corruption and healthcare access. In many healthcare systems, social capital plays a pivotal role in shaping interactions between patients and providers, but it can also foster corrupt practices like favouritism and exclusion. For instance, Transparency International highlighted that favouritism in healthcare delivery, where providers prioritise patients with whom they have personal or social connections, can result in unfair access to services.^2^ Patients with stronger networks can bypass official processes, such as waiting times or even making informal payments. In contrast, those without such connections, particularly from rural or low socioeconomic backgrounds, are systematically excluded from receiving care.

Thus, in contexts like Afghanistan, Uganda and Bosnia, social ties were found to be more effective than bribes, in securing care, with well-connected patients able to jump queues or access better treatment.^19 20^ This misuse of social capital worsens inequalities by undermining the equity of healthcare provision. As shown in sub-Saharan Africa, ethnic and social favouritism can lower infant mortality rates for connected groups while leaving marginalised populations without adequate care.^21^ Thus, while social capital can foster trust and mutual aid, it can also become a tool for corruption, perpetuating inequitable healthcare access, a significant barrier to achieving UHC.

This evidence illustrates that social capital, when concentrated in informal networks, can potentially facilitate corruption by creating systems of exclusion and favouritism. In these systems, access to healthcare reflects connections rather than need, amplifying existing inequalities.

These findings underscore the importance of governance and anticorruption strategies that fully engage with the context to ensure effective healthcare delivery and progress towards UHC, particularly in terms of social relationships. However, as previous studies from Nigeria have shown, efforts to combat corruption through conventional governance and anticorruption measures have yielded limited success,^3^ this suggests the need for alternative approaches. This paper hypothesises that social capital within the Nigerian health sector can worsen corruption by misusing personal networks, seeking to inform more nuanced policy interventions to combat corruption and advance UHC.

## Research methods

### Study design

Data were obtained from a cross-sectional study in two Nigerian states. One in southeastern Nigeria and the other in the northwestern part of Nigeria. In each state, data were collected from one urban, two semiurban and two rural local government areas (LGAs). Since the study covered two states, this resulted in a total of six LGAs: two urban, four semiurban and four rural.

### Sampling and data collection

The study population consisted of households in the selected local governments of the two selected states. Respondents had lived in the community for at least 6 months and were between 18 and 65 years old. In the past 6 months, they sought care in a public healthcare facility for a dependent, themselves or another adult. The 6 months benchmark was erected to minimise recall bias. Exclusion criteria were medically unfit individuals, household members with inadequate information about their health consumption and those who only sought care in the private health sector. Government areas of the two selected states. A total of 1659 households were selected and interviewed in the survey. When more than one family member had sought care, the most recent visit was captured for that household to ensure better recall.

Households were selected using a purposive multistage sampling method. The first stage involved selecting two states perceived as secure to ensure ease of data collection. In the second stage, LGAs were grouped and chosen to capture urban, semiurban and rural locations, where at least three LGAs were selected in each state. The last stage involved selecting households based on those who accessed care from a public facility within the previous 6 months to the interview date.

However, given this multistage approach, respondents within the same LGA were likely to share similar contextual factors. To address this, all regression analyses were adjusted for clustering at the LGA level, thereby correcting for potential underestimation of SEs due to intracluster correlation.

Data were collected using a pretested questionnaire, whose initial draft had been adapted from a previously published study. It was administered by an interviewer to reduce information bias.

#### Pairs of interviewers

One entered data on a tablet and the other on paper. The tablet version required each question to be answered to proceed to the next (although with skips where certain responses rendered subsequent questions non-applicable), and the two versions were compared at the end of the interview to minimise errors. Interviewers were research assistants selected based on having from those who had completed at least secondary education and the ability to who could speak the local dialect of the data collection location. Depending on the area, interviews were conducted in English, Hausa, Igbo and/or Pidgin languages were used for the data collection.

Data were collected on respondents’ sociodemographic characteristics, measures of social capital and healthcare-seeking patterns. See online supplemental material 2 for the survey questionnaire.

### Patient and public involvement

Patients or the public were not involved in the design, conduct, reporting or dissemination plans of the research.

### Operationalisation of constructs

Social capital: Social capital was operationalised using multiple indicators reflecting household and individual connections within social, political and community networks relevant to accessing healthcare. Specifically, indicators included membership in religious organisations, professional associations, political parties or Ward Development Committees (WDC), among others.

These indicators were measured as binary variables (active member=1, non-member/inactive=0). To summarise these dimensions into a single measure, a social capital index was derived using factor analysis (FA), capturing the essential features of household social capital relevant to healthcare access. In using this method, we adopted the principal component analysis (PCA) section, which is used at the extraction stage especially when the variables used are binary like in our case where active member=1, non-member/inactive=0.^22^,^25^ We used the PCA method to extract the information used to create the index called the social capital index, which was used as an independent variable in our regression model. The primary purpose of using this method was to create an index, which would be a continuous variable suitable for analysis in this study. See online supplemental material 1 for full results of the PCA, including eigenvalues, factor loadings and uniqueness values, presented in online supplemental tables 1 and 2. It is important to note that the FA using the PCA extraction method was only used in this study to create the index variable capturing essential parts of the variables used to capture social capital.

Corruption: Corruption was operationalised through service users’ engagement in behaviours to secure healthcare in public facilities, including paying unapproved fees (with or without using personal connections) and using personal connections to bypass official processes.

Responses were coded 1 if respondents reported any such behaviour and 0 otherwise.

### Data analysis

Frequencies, percentages, mean and SD were used to present the sociodemographic characteristics of the respondents. Analyses were conducted using complete cases; participants with missing key variables were excluded from the analysis. The primary dependent variable for our analysis was whether a respondent reported engaging in various behaviours to access healthcare in a public facility. Specifically, whether they responded ‘yes’ to have done the following to access care at a public facility paid unapproved payment with or without using connections to secure treatment, or using personal connections to secure treatment, with the variable coded 1 if respondents answered yes to any options and 0 otherwise. Associations between reporting any of these behaviours and key household and individual respondent characteristics were done using cross-tabulations and multivariate binary logistic regression. The independent variables included in the regression models were selected based on the previous literature, showing an association with engaging in corruption while receiving care, while gender, age and years of completed education of the household member were added as control variables. We collected data on several indicators of social capital (membership of religious organisations, political parties, WDC (or having powerful family or household members)). These variables were compressed to one variable using factor analysis. Through this, a factored value (0 and 1) capturing the essential features of the social capital variables was created (called the social capital index).

Table 1 shows the expected results from the logistic regression. Membership in a religious organisation, political party, WDC or professional association were coded as either active or inactive membership or not a member. Household ownership of social capital was expected to have a positive relationship with corruption, since having strong connections and networks may increase the likelihood of engaging in it. This variable was coded as 0 if the household lacked such connections, and 1 if they had them.

**Table 1:** Included variables and a priori expectation of impact on engaging in corrupt acts while receiving care in public facilities

Variables	Nature of data	Expected sign
Social capital index	Continuous	Positive (+)
Asset quintile	Ordinal	Positive (+)
Gender (female)	Nominal/binary	Negative (−)
Age	Continuous	Positive (+)
Years spent on formal education	Continuous	Positive (+)

We assessed potential multicollinearity among explanatory variables (including education and social capital index) using variance inflation factors (VIFs). All predictors recorded VIF values below 3.0, well under the conventional threshold of 5.0, indicating that multicollinearity was not a concern. Thus, coefficient estimates can be interpreted with confidence.

The continuous variables in the study (social capital, age and years spent on formal education) were expected to produce a positive relationship with increased corruption. For gender, males were expected to engage in more corrupt activities than females, while receiving care based on available corruption studies, which showed that men engage in corrupt practices more often than women.^26 27^ Furthermore, in the Nigerian setting, given that male household members more often take on the role of family providers, they are more likely to face and engage in corruption in daily activities.^27^

### Findings

The general characteristics of the sample are shown in table 2. Briefly, out of the 1659 respondents, 60.3% were female. More than 80% of the participants were educated, and more than 75% lived with their spouses. Most of them (78.7%) have also experienced one form of disorderliness or corruption while accessing care in health facilities.

**Table 2:** Demographic characteristics of the respondents

Variable	Frequency	Percentage	Mean (SD)	Engaged in corruption (%)
Age			37.39 (12.7)	
Gender				
Female	1000	60.3		39.9
Male	659	39.7		64.3
Household size			8.07 (6.91)	
Education level				
I didn’t go to school	144	8.7		7.7
Primary	146	8.8		9.8
Junior secondary	89	5.4		5.1
Senior secondary	702	42.3		39.5
University	242	14.6		15.2
Polytechnics	130	7.8		10
Educational colleges	162	9.8		11.3
Others	44	2.7		1.5
Marital status				
Single (never married)	186	11.2		12
Living with spouse	1255	75.6		71
Divorced/separated	37	2.2		3.3
Widowed	181	10.9		13.7
Type of public facility visited.				
District/general/cottage hospital	552	33.3		41.7
Health post	42	2.5		2.8
Primary healthcare	857	51.7		39.5
Tertiary hospital	206	12.4		15.8
Don’t know	1	1		0.1
Other	1	1		0.1

Table 3 outlines the experience of respondents accessing care and the proportion reporting using family or personal connections and other means to secure access to treatment, which shows that a greater percentage of those who used connection to access healthcare services used it to secure treatment.

**Table 3:** Respondents’ experiences when seeking care

Variable	Frequency	Percentage
Experiences while receiving healthcare		
One or more persons jumping the queue	495	29.8
Asked to bring an excessive amount of consumables	160	9.6
Paid higher fees than the advertised price	125	7.5
Unavailability of health workers	224	13.5
The facility was closed when it should have been open	70	4.2
Provided favour to health workers before receiving care	34	2
Health workers refused to provide care	200	12.1
Received help from the following people to obtain service from the health facility		
Powerful family members	816	49.2
Powerful close friends or friends with connections to powerful people	467	28.1
Powerful people living in your community	158	9.5
Powerful members of community organisation	43	2.6
Powerful people at the district or LGA	18	1.1
Godfather/godmother	48	2.8
Powerful people at the national level (politicians)	23	1.4
Using the connection to receive care for the last 6 months		
No payment and no connections were used	1152	69.4
No payment, but I/we used connections to secure treatment	195	11.8
Not sure/don’t know	126	7.6
Paid unapproved/extra payment (to staff), and I/we also used connections to secure treatment	79	4.8
Paid unapproved/extra payment (to staff), but no connections were used	107	6.4
Kind of connections		
Householder members	53	3.2
Close family	96	5.8
Extended family (including the spouse’s family)	77	4.6
Close friends	82	4.9
People living in your community	72	4.3
Community organisation	29	1.7
Powerful people living in your community	24	1.4
Politicians	4	0.2
People in government (eg, district, LGA)	44	2.7
Engagement in corruption		
Engaged in corruption	49.6	823
Did not engage in corruption	50.4	836

LGA, local government area.

Table 4 shows that active members of religious organisations were slightly and significantly (p<0.05) less likely (47.1%) to engage in corrupt practices in health facilities. In comparison, active members of political parties (71.5%), community governance structures such as the WDC members (73%) and members of professional associations (72.2%) are significantly (p<0.01) more likely to engage in corruption to receive health services.

**Table 4:** Organisation membership and engagement in corrupt practices

Organisation	Pooled result			Southeastern state result			Northwestern state result		
	Engaged in corrupt practices	Did not engage in corrupt practices	χ^2^ (p value)	Engaged in corrupt practices	Did not engage in corrupt practices	χ^2^ (p value)	Engaged in corrupt practices	Did not engage in corrupt practices	χ^2^ (p value)
Religious organisation			7.4951 (0.024)			0.5646 (0.754)			33.4889 (0.000)
Active member	403 (47.1%)	453 (52.9%)		204 (33.66%)	402 (66.34%)		200 (79.37%)	52 (20.63%)	
Inactive member	88 (46.8%)	100 (53.2%)		45 (36.59%)	78 (63.41%)		286 (58.37%)	204 (41.63%)	
Not a member	331 (54%)	282 (46%)		37 (36.27%)	65 (63.73%)		51 (59.30%)	35 (40.70%)	
Political parties at the community level			67.7591 (0.000)			5.4327 (0.066)			38.2259 (0.000)
Active member	173 (71.5%)	69 (28.5%)		29 (45.31%)	35 (54.69%)		144 (80.90%)	34 (19.10%)	
Inactive member	103 (59.2%)	51 (40.8%)		223 (32.70%)	459 (67.30%)		325 (57.83%)	237 (42.17%)	
Not a member	547 (44%)	696 (56%)		34 (40.00%)	51 (60.00%)		68 (77.27%)	20 (22.73%)	
Ward Development Committee			89.8816 (0.000)			2.8688 (0.238)			36.7813 (0.000)
Active member	157 (73%)	58 (27%)		16 (40.00%)	24 (60.00%)	34 (	141 (80.57%)	34 (19.43%)	
Inactive member	88 (71.5%)	35 (28.5%)		260 (33.72%)	511 (66.28%)		317 (57.74%)	232 (42.26%)	
Not a member	578 (43.8%)	743 (56.2%)		10 (50.00%)	10 (50.00%)		79 (75.96%)	25 (24.04%)	
Professional association			90.8610 (0.000)			19.5777 (0.000)			54.3442 (0.000)
Active member	143 (72.2%)	69 (28.5%)		44 (50.57%)	43 (49.43%)		99 (89.19%)	12 (10.81%)	
Inactive member	91 (75.8%)	51 (40.8%)		224 (31.46%)	488 (68.54%)		365 (58.03%)	264 (41.97%)	
Not a member	589 (43.9%)	696 (56%)		18 (56.25%)	14 (43.75%)		73 (82.95%)	15 (17.05%)	

Disaggregating the results by state, we discovered that active religious organisation members in the southeastern state insignificantly (p>0.05) were less likely to engage in corrupt practices (33.66%). The northwestern state shows that active religious organisation members are significantly (p<0.01) more likely to engage in corrupt activity while receiving care (79.37%). As to being an active political party member, those in the southeastern state were not significantly more or less likely to engage in corrupt practices (45.31%), unlike those in the northwestern state who were significantly (p<0.01) more likely to engage in the corrupt act (80.9%).

Household members being active members of WDC in the southeastern state were neither more nor less likely to engage in corrupt practices (40%), unlike those in the northwestern state who were significantly (p<0.01) more likely to engage in corrupt acts (80.57%). As for being an active professional association member, the results show that in the southeastern state, they were significantly (p<0.05) slightly more likely to engage in corrupt acts while receiving care (50.57%). In contrast, the northwestern state was overwhelmingly and significantly (p<0.01) more likely to engage in corrupt acts (89.19%).

Table 5 presents the pooled and disaggregated logistic regression results on predictors of corruption during healthcare utilisation in public facilities. In the pooled analysis, households with higher social capital were more than two times as likely to engage in corrupt practices while receiving care (OR=2.24, p<0.01). Wealth was also a significant predictor, as households in the extremely rich quintile had 66% higher odds of engaging in corrupt behaviour compared with the poorest households (OR=1.66, p<0.01). Female household members had substantially lower odds of engaging in corrupt practices relative to males (OR=0.45, p<0.01). Age was positively associated with corruption, with each additional year increasing the odds by about 1% (OR=1.01, p<0.01). Years of formal education were not significantly associated with corruption in the pooled model.

**Table 5:** Influence of social capital on corrupt behaviour during care in public facilities

Variables	Pooled result OR (p value)	Southeastern state result	Northwestern state result
	OR (P>|t|)		
Used social capital to access care	2.24 (0.00)*	1.16 (0.63)	2.04 (0.00)*
Asset ownership quintile			
Poor	1.21 (0.24)	1.58 (0.04)*	0.72 (0.18)
Average	1.18 (0.34)	1.24 (0.35)	0.89 (0.66)
Rich	1.26 (0.19)	1.18 (0.50)	0.97 (0.92)
Extreme rich	1.66 (0.00)*	1.30 (0.33)	1.22 (0.42)
Gender (female)	0.45 (0.00)*	0.75 (0.07)	0.35 (0.00)*
Age	1.01 (0.00)*	1.04 (0.19)	1.02 (0.00)*
Years spent on formal education	1.06 (0.50)	1.05 (0.02)*	1.01 (0.90)
Prob>χ^2^	0.00*	0.00*	0.00*
Pseudo R^2^	0.11	0.02	0.10

* Significant at 5%.

The state-level analyses reveal important differences. In the Southeastern state, none of the social capital, wealth, gender or age variables were significantly associated with corruption. However, education emerged as a predictor: each additional year of formal schooling was associated with a 5% increase in the odds of engaging in corrupt practices (OR=1.05, p<0.05).

By contrast, several factors were significant in the Northwestern state. Households with higher social capital had approximately two times the odds of engaging in informal payments (OR=2.04, p<0.01). Poor households, compared with the extremely poor, were more likely to engage in corrupt practices (OR=1.58, p<0.05). Gender effects were pronounced, as females had 65% lower odds of engaging in corruption relative to males (OR=0.35, p<0.01). Age was also significant, with each additional year increasing the odds of corruption by 2% (OR=1.02, p<0.01). Furthermore, years of formal education were associated with a 5% increase in the odds of corruption (OR=1.05, p<0.05).

## Discussion

This paper sought to explore the interplay of social capital to corrupt practices in health facilities in Nigeria. The findings show that social capital is significantly associated with the prevalence of corruption in healthcare services in a southern and a northern state of Nigeria. The influence of social group associations on corruption in the health system aligns with findings from Nieminen *et al* in 2013, which highlighted the role of social networks and participation in improving access to healthcare services. Although mechanisms behind this relationship are unclear.^28^

The findings of this study indicate that individuals often relied on close family and friends to access healthcare services, aligning with literature that highlights social capital as an important facilitator of healthcare access.^29^ Although our study did not directly assess financial outcomes, prior research suggests that social capital can contribute to the mitigation of financial burdens by mobilising support, pooling resources and lessening reliance on formal service expenditures.^30^ Odii *et al*’s study provides a deeper understanding of how social capital influences actor behaviour within Nigeria’s healthcare system, particularly through power dynamics, political connections and kinship ties. Their findings suggest that individuals with strong social networks, primarily political or familial influence, are more likely to engage in corrupt practices to access healthcare services. In contexts, where medical resources are scarce, people leverage these connections to bypass formal systems, gaining preferential access to ensure adequate to care—however, this is often at the expense of care provided to others.

Our findings indicate that affiliation with professional associations and political parties was found to promote corrupt activities, while membership in religious organisations tends to reduce them. This may be due to the varying levels of reach and influence individuals in these organisations possess and their ability to leverage this influence to achieve specific objectives. A study done in Nigeria describes how health workers may be absent from duty with impunity if their absence is protected by powerful connections, undermining the system’s efficiency and fairness.^15^

Education and wealth influenced engagement in corrupt practices. Higher levels of education and wealth enable individuals to leverage social networks more effectively, increasing their opportunities to engage in corrupt practices. This aligns with the report made by the United Nations Office on Drugs and Crime, which reported that education influences both the likelihood of encountering and rejecting corrupt practice.^27^

A study on the COVID-19 pandemic highlighted that social capital played a critical psychosocial role in accessing healthcare when resources were limited. During the pandemic, the global strain on medical supplies and services, exacerbated by an ageing population and increased demand for health services, intensified the competition for care. In this context, social capital, such as connections and networks, became as important as medical factors in determining access to care.^31^ Many patients turned to personal connections, seeking out health providers they knew to help navigate the overwhelmed healthcare system.^32 33^ This study’s focus on two Nigerian states provides valuable insights into the role of social capital in healthcare-related corruption but is limited by its regional scope. The exclusion of individuals who did not seek healthcare also means that the full spectrum of challenges faced by different groups remains unexplored. Nonetheless, the findings demonstrate that social capital significantly influences corrupt practices in healthcare access, with organisational membership emerging as a key factor.

Furthermore, the social capital index, derived from binary indicators through exploratory factor analysis, may have simplified complex dimensions of community networks. While the state-level focus enabled valuable insights into local patterns, it may mask rural–urban and socioeconomic variations within states. The logistic regression models also yielded low pseudo-R² values (0.02–0.11), a common occurrence in social science research, yet the models remain informative given significant likelihood ratio tests and theoretically consistent findings. Moreover, defining corruption as a binary composite of informal payments, use of connections, and gift-giving may have reduced nuance; alternative definitions might alter results, underscoring the need for sensitivity analyses. Finally, the study did not test for interaction effects, such as whether education moderates the relationship between social capital and corruption. Addressing these issues in future research would provide a richer understanding of how social capital shapes healthcare access and governance outcomes. In light of these findings, policymakers must design and implement strategies that enhance transparency and accountability within the healthcare system. Efforts should focus on reducing the influence of informal networks that perpetuate corruption while ensuring that access to healthcare is based on need rather than connections. Additionally, addressing regional disparities in healthcare access should be a priority, as local contexts play a significant role in shaping how social capital impacts healthcare services.

Further research, particularly involving a broader range of regions across Nigeria, is needed to fully understand the dynamics of social capital in healthcare and its role in corrupt practices. Combining quantitative and qualitative approaches will provide a more comprehensive understanding and inform targeted, evidence-based public health policies to reduce corruption and promote equitable healthcare access for all Nigerians.

## Supplementary material

10.1136/bmjgh-2025-020195 online supplemental file 1

10.1136/bmjgh-2025-020195 online supplemental file 2

## Data Availability

Data are available upon reasonable request.
