# Supplementary material for: Contribution and influence of social capital on corruption in the health sector: a view through the lens of service users
Source: BMJ Glob Health. 2025 Dec 9;10(12):e020195. doi: 10.1136/bmjgh-2025-020195 (PMC12699593; doi:10.1136/bmjgh-2025-020195)
Supplement: online supplemental file 1 [file bmjgh-10-12-s001.docx]

**Supplemental material 1: Principal Component Analysis results**

The uniqueness values showed that being member of political party at the community and LGA levels were well represented by the factors, whereas others, such as membership of professional association and facility health committee, did not align as closely with the main factor patterns.

Finally, the social capital index derived from these components showed a wide distribution, with a mean near zero and values ranging from -3.32 to 0.77, reflecting significant variation in social capital ownership across the surveyed population.

**Supplemental Table 1: Principal Component Analysis Results**

| **Factor** | **Eigenvalue** | **Difference** | **Proportion** | **Cumulative** |
| --- | --- | --- | --- | --- |
| Factor1 | 3.27586 | 2.00638 | 0.4680 | 0.4680 |
| Factor2 | 1.26948 | 0.44650 | 0.1814 | 0.6493 |
| Factor3 | 0.82298 | 0.23518 | 0.1176 | 0.7669 |
| Factor4 | 0.58780 | 0.05400 | 0.0840 | 0.8509 |
| Factor5 | 0.53380 | 0.16486 | 0.0763 | 09271 |
| Factor6 | 0.36894 | 0.22781 | 0.0527 | 0.9798 |
| Factor7 | 0.14113 | - | 0.0202 | 1.0000 |

LR Test: Independent vs. Saturated Model

Chi^2^(21) = 4775.31, Prob > Chi2 = 0.0000

**Supplemental Table 2: Factor Loadings (Pattern Matrix) and Unique Variances**

| **Variable** | **Factor1** | **Factor2** | **Uniqueness** |
| --- | --- | --- | --- |
| Religious organisation | 0.3862 | 0.6514 | 0.4265 |
| Political party at the community level | 0.8338 | 0.2500 | 0.2422 |
| Political party at the LGA level | 0.8377 | -0.2929 | 0.2125 |
| Professional association | 0.6932 | 0.1047 | 0.5085 |
| Ward Development Committee | 0.7841 | -0.2162 | 0.3384 |
| Heath facility committee | 0.7342 | 0.0729 | 0.4557 |
| Women group | 0.3087 | 0.7961 | 0.2709 |

Summary Statistics of Asset Index based on 1652 observations: mean -2.52; Standard deviation 1; minimum -3.32; maximum 0.77
