# Supplementary material for: Contribution and influence of social capital on corruption in the health sector: a view through the lens of service users
Source: BMJ Glob Health. 2025 Dec 9;10(12):e020195. doi: 10.1136/bmjgh-2025-020195 (PMC12699593; doi:10.1136/bmjgh-2025-020195)
Supplement: online supplemental file 2 [file bmjgh-10-12-s002.docx]

**Supplementary material 2: Study Questionnaire**

**ACCOUNTABILITY IN ACTION PROJECT**

**Inefficiencies, distortions and corruption in provision of healthcare services:**

**SURVEY QUESTIONNAIRE**

**Introduction**

Dear respondent,

We are researchers from the Health Policy Research Group, College of Medicine, University of Nigeria, Enugu. We are interested in your opinion on your experiences in using healthcare services, what happened to you, how were you treated and in the obstacles you may have faced. We are also interested in your opinion about the behaviours of different healthcare providers in your community and how to improve the quality and fairness of the services provided. To achieve this aim, we wish to ask you a number of detailed questions. You shall in the future get the results of this exercise, which we hope to use to help your community and the government to improve access to healthcare services through improvement of the efficiency of services and elimination of unethical and corrupt practices in health facilities. All information given will be confidential. Your participation is voluntary, and you do not have to answer questions you do not want to answer and you can terminate the interview at any time.

***Instruction: Please fill the next section at the beginning and at the end of the interview.***

**Unique ID follows this formula: 0X-YYYY**

**X equals to 1 = Enugu; 2 = Kano; 3 = Malawi**

**YYYY starts from 0001 to 9999 and it should be unique in each location (ie Enugu, Kano, Malawi)**

**For example: 02-0024 gives us information that this is the 24th respondent from Kano.**

**It is possible to have 01-0024 in Enugu and 03-0024 in Malawi**

Unique Respondent ID:

What time did the interview start: __________________________?

Gender: Male / Female

Name of the interviewer ___________________________________________________

Date of interview < / / >

Interview location: [ ] 1 = Enugu 2 = Kano 3 = Malawi

Name of administrative divisions:

LGA (Nigeria) or district (Malawi)________________________

Name of ward (Nigeria)

GPS coordinate _________________________

Geographic type of interview location: [ ] 1 = urban 2 = semi/peri-urban 3 = rural

*Note to interviewer: Urban area is defined as area with the presence of financial institution (such as Bank) and government offices, active commercial activity, good road networks, or where the LGA headquarter is located. Peri/semi urban is located on the outskirts of large urban areas but retain rural characteristics such as reliance on agricultural production.*

*Important notes****:***

1. *We will only conduct interview in households where* ***at least one member sought care in public facilities****. If more than one member of household visited health facility, select the person who has* ***the most recent visit and is at home****.*
2. *The definition of a* ***household*** *is a person or group of related or unrelated persons who live together in the same dwelling unit(s), who acknowledge one adult male or female as the head of the household, who share the same housekeeping arrangements and who are considered a single unit.*
3. *The respondent must have personally used health services or sought care for a dependent (for example: child) or another adult household member (e.g., taken them to facilities, paid, bought drugs) in the past 6 months from the date of the survey. If the respondent is too old or too ill to respond, ask the main respondent to provide information (the same situation as it they were dependent).*
4. *If someone else in the household sought care but the respondent was not responsible for them and doesn’t know details about their care, attempt to interview that household member or select another household.*
5. *If someone else in the household sought care but either was too ill to answer or a minor (<17 year old), attempt to interview their carer/guardian or anyone who knows more detail about their care.*
6. *The respondent should be interviewed only if the care process they are talking about is in* ***the public health sector****, even if they have used the private sector as well. They should be excluded if they have used only private services.*
7. *After the screening questions, this survey is divided into five sections:*
   1. *Health seeking and experience of rule-breaking/corruption*
   2. *Vignettes*
   3. *Perceptions*
   4. *Complaints process*
   5. *Demographic and socioeconomic information (we mostly get information on the household level but there are some individual level data such as education)*
8. *If the respondent provide information about themselves in section I, they answers all other questions for themselves (including section V about SES).* ***A few important exceptions*** *apply:*
   1. *If in section I the respondent provides information about a child, dependent or an old person who cannot answer, the respondent should still provide their own answers in section II–V.*
   2. *If in section I the respondent provides information about another adult whom they helped but the other person made all decision related to the visit/s, the respondent should provide their own answer in section II–IV, except for a few personal characteristics in section V (ie they should report the personal characteristics of the adult whose experience is captured in section I)*

**Screening questions**

*INTERVIEWER: Please ask all screening questions before the rest of the interview*

1. Have you lived in this community for at least 6 months?
   1. Yes Go to Q2
   2. No – terminate interview
   3. Not sure/can’t remember Go to Q2
2. In the **past 6 months**, have you (or your household member) visited a **public health facility** (at any level – primary, secondary, or tertiary) for any reason related to health? (e.g., seek care for illness, pregnancy, vaccination, health certificate, etc.)

*INTERVIEWER: They could have gone to facilities because to seek care (treatment or preventive services such as immunisations, pregnancy, and check-ups), to get certificates or for any other reason. It doesn’t matter if the most recent visit wasn’t at public health facility.*

- 1. Yes GO TO Q4 in section I
  2. No GO TO Q3
  3. Not sure/can’t remember

1. If you or someone from your household **didn’t go to public health facility for any reason** in the past 6 months, what did you do?

*Interviewer: this question is aimed to understand any reason why people didn’t go to public facility, whether they sought care somewhere else or didn’t fall ill or didn’t have any need to go for prevention care or health certificate.*

1. The condition/ailments were not serious enough to require seeking care at a facility/ will improve through self-treatment
2. Healthcare facility is located too far away
3. Health care services are of poor quality
4. There are abuses, demands for unapproved payments by staff in public healthcare facilities I want to avoid
5. I/they could not afford to pay for the drugs or treatment
6. Staff is more often present in private facilities so I (they) prefer to go there
7. No time/ I/they cannot take time off work
8. Other (specify) ______________________
9. Not sure / Don’t know

TERMINATE INTERVIEW

• Aunt/uncle

• Cousin

• Niece/Nephew

• Other_________________specify

 Who made the decision in relation to the visit to health facility?

• Myself

• The other person

• Not sure / Don’t know

Instruction: The whole questions below are for the one who sought care

5. In what month did you last visit a public health facilities (for care or other health related reasons) ?

______________month (January-December, please specify)

Not sure / Don’t know

6. What was it for? (Only choose one answer – the primary reason)

• Pregnancy/Childbirth related

• Immunisation

• Family planning

• Infectious disease with fever (e.g. Malaria, Typhoid, etc.)

• Long-term non-communicable condition (e.g. diabetes, hypertension, cardiovascular disease)

• Trauma/injury

• Surgery (for other than the above listed reasons)

• Mental health (depression, drug use or other problems)

• Dental

• Obtaining a certificate

• Asking for a referral to hospital

• Other (specify) _______________________

• Not sure/ Don’t know

NOTE FOR INTERVIEWER: the following questions are about this particular occasion mentioned in Q6, with one or multiple visits to facilities. Keep the respondent focused on that health problem/reason and do not mix up visits belonging to different health problem

7. For this latest problem, how many times did you/other member of household need to go to public health facilities?

________ specify number of visits (as best remembered)

8. For this latest problem, did you have to stay overnight?

• Yes

• No

9. For this latest problem, what type of public facility did you go to (latest occasion in the past 6 months)? (just one option)

• Health Post

• Primary Health Care Centre

• General/Cottage/District hospital

• Tertiary hospital (Nigeria)

• Other (specify) _______________________

• Not sure/ Don’t know

10. What were the main reasons you went there (latest occasion in the past 6 months)? (select up to 2 answers)

• Close to where you live/convenient

• Staff attitude is good/friendly

• No need to pay or very small payments are required

• Advised by friend/relative to go there to interact with other people with similar situation (e.g. pregnant women)

• I know the staff working there

• I have a family member working there

• Drugs are always available

• The facilities have higher quality of care than others in the area

• Other (specify) _______________________

11. Who did you see (think of all visits related to the most recent health problem in the past 6 months)? (Tick all applicable)

• Doctor

• Medical officer

• Nurse/ nursing assistant

• Midwife

• Community health extension worker

• Junior CHEW

• Volunteer or ad hoc staff member (medically qualified)

• Traditional healer

• Other type of staff __________________(specify)

• Not sure/ Don’t know

12. In what type of facilities did you seek care apart from public facility? (tick all applies)

• Private

• Non-governmental (faith-based facility/mission hospital)

• Individual provider, not in health facility (e.g. traditional)

• I only sort care in public facilities

• Traditional Healer

• None, I only sought care in public facility (Skip Q13)

• Other __________________(specify)

• Not sure/ Don’t know

13. If you have started using different types of facilities apart from public, why is this? (Up to 2 answers)

• Private facilities provide higher quality service

• Doctor or health workers in public facilities referred me to his/her private clinic

• Waiting time in the public facilities are too long

• Staffs are more often available in private facilities

• Private facilities are closer to my home

• There is charging in public facilities that I want to avoid

• Other (specify)

• Not sure/ Don’t know

Broad brush of rule-breaking experience

14. Did you or your family member experience any of the following when receiving services or attending a public facility for a different reason: (tick all applies) Please make sure that they are still talking about the most recent occasion/reason

• One or more persons jumping the queue (e.g. obtaining priority service because they know staff)

• You were asked to bring excessive amounts of consumables

• You had to pay higher fees than the advertised price for services

• Few or no health workers were available to provide services

• Facility was closed when it should be opened

• You had to provide favours or services to facility or health worker to get medical attention

• Health workers were free but refused attending to you

• None of these

• Not sure/ Don’t know

15. Did you observe any of those things happening to others? (tick all applies) Please make sure that they are still talking about the most recent occasion/reason

• One or more persons jumping the queue (e.g. obtaining priority service because they know staff

• They were asked to bring excessive amounts of consumables

• You had to pay higher fees for services

• Few of no health workers were available to provide services

• Facility was closed when it should be opened

• People were asked to provide favours or services to facility or health worker

• Health workers were free but refused attending to others

• None of these

• Not sure/ Don’t know

Health expenditure including informal payments

We will now ask you about payments or the value of in-kind contributions/gifts for any items that you made during your visit/s (latest occasion in the past 6 months). We want to focus on the most recent occasion regardless of when it happened within the six months period. If you made multiple visits for the same occasion/reason, please focus on the total payments for the whole occasion of seeking care.

[Interviewer: ensure people focus on the same occasion of seeking care, providing information about total across all visits and don’t mix up payments for visits belonging to different occasion]

16. Did you have to pay or contribute something for consultation?

• Yes, I paid it to a health worker inside the facility. Go to Q16a

• Yes, I paid it at a facility pharmacy. Go to Q16a

• Yes, I paid it at a shop or staff just outside the facility. Go to Q16a

• No, I didn’t pay at all. Go to Q17

a. How did you pay or contribute?

 Paid by cash.

 Paid by electronic transfer.

 Paid by in-kind contribution (demanded/expected by the health worker)

 Gave gift.

b. How much was the total cost of any payment or in-kind contribution/gift for this item? _____________Naira/Kwacha

c. Did you think this payment were approved/official?

 I know that they are approved/official

 I know that they are non-approved/non-official

 I don’t know

d. Did you get the receipt?

 Yes and what I paid matched with the receipt

 Yes, but I paid more than the receipt

 No, I didn’t get any receipt

17. Did you have to pay or contribute something for drugs?

• Yes, I paid it to a health worker inside the facility. Go to Q17a

• Yes, I paid it at a facility pharmacy. Go to Q17a

• Yes, I paid it at a shop or staff just outside the facility. Go to Q17a

• No, I didn’t pay at all. Go to Q18

a. How did you pay or contribute?

 Paid by cash.

 Paid by electronic transfer.

 Paid by in-kind contribution (demanded/expected by the health worker)

 Gave gift.

b. How much was the total cost of any payment or in-kind contribution/gift for this item? _____________Naira/Kwacha

c. Did you think this payment was approved/official?

 I know that they are approved/official

 I know that they are non-approved/non-official

 I don’t know

d. Did you get the receipt?

 Yes and what I paid matched with the receipt

 Yes, but I paid more than the receipt

 No, I didn’t get any receipt

18. Did you have to pay or contribute something for consumables/supplies?

• Yes, I paid it to a health worker inside the facility. Go to Q18a

• Yes, I paid it at a facility pharmacy. Go to Q18a

• Yes, I paid it at a shop or staff just outside the facility. Go to Q18a

• No, I didn’t pay at all. Go to Q19

a. How did you pay or contribute?

 Paid by cash.

 Paid by electronic transfer.

 Paid by in-kind contribution (demanded/expected by the health worker)

 Gave gift.

b. How much was the total cost of any payment or in-kind contribution/gift for this item? _____________Naira/Kwacha

c. Did you think this payment was approved/official?

 I know that they are approved/official

 I know that they are non-approved/non-official

 I don’t know

d. Did you get the receipt?

 Yes and what I paid matched with the receipt

 Yes, but I paid more than the receipt

 No, I didn’t get any receipt

19. Did you have to pay or contribute something for diagnostic tests?

• Yes, I paid it to a health worker inside the facility. Go to Q19a

• Yes, I paid it at a facility pharmacy. Go to Q19a

• Yes, I paid it at a shop or staff just outside the facility. Go to Q19a

• No, I didn’t pay at all. Go to Q20

a. How did you pay or contribute?

 Paid by cash.

 Paid by electronic transfer.

 Paid by in-kind contribution (demanded/expected by the health worker)

 Gave gift.

b. How much was the total cost of any payment or in-kind contribution/gift for this item? _____________Naira/Kwacha

c. Did you think this payment were approved/official?

 I know that they are approved/official

 I know that they are non-approved/non-official

 I don’t know

d. Did you get the receipt?

 Yes and what I paid matched with the receipt

 Yes, but I paid more than the receipt

 No, I didn’t get any receipt

20. Did you have to pay or contribute something for certificates or other administrative charges?

• Yes, I paid it to a health worker inside the facility. Go to Q20a

• Yes, I paid it at a facility pharmacy. Go to Q20a

• Yes, I paid it at a shop or staff just outside the facility. Go to Q20a

• No, I didn’t pay at all. Go to Q21

a. How did you pay or contribute?

 Paid by cash.

 Paid by electronic transfer.

 Paid by in-kind contribution (demanded/expected by the health worker)

 Gave gift.

b. How much was the total cost of any payment or in-kind contribution/gift for this item? _____________Naira/Kwacha

c. Did you think this payment were approved/official?

 I know that they are approved/official

 I know that they are non-approved/non-official

 I don’t know

d. Did you get the receipt?

 Yes and what I paid matched with the receipt

 Yes, but I paid more than the receipt

 No, I didn’t get any receipt

21. Did you have to pay to jump the queue?

• Yes, I paid it to a health worker inside the facility. Go to Q21a

• Yes, I paid it at a facility pharmacy. Go to Q21a

• Yes, I paid it at a shop or staff just outside the facility. Go to Q21a

• No, I didn’t pay at all. Go to Q22

a. How did you pay or contribute?

 Paid by cash.

 Paid by electronic transfer.

 Paid by in-kind contribution (demanded/expected by the health worker)

 Gave gift.

b. How much was the total cost of any payment or in-kind contribution/gift for this item? _____________Naira/Kwacha

22. Did you have to pay to something but not sure for what item/service?

• Yes, I paid it to a health worker inside the facility. Go to Q22a

• Yes, I paid it at a facility pharmacy. Go to Q22a

• Yes, I paid it at a shop or staff just outside the facility. Go to Q22a

• No. Go to Q23

a. How did you pay or contribute?

 Paid by cash.

 Paid by electronic transfer.

 Paid by in-kind contribution (demanded/expected by the health worker)

 Gave gift.

b. How much was the total cost of any payment or in-kind contribution/gift for this item? _____________Naira/Kwacha

c. Did you think these payments were approved/official?

 I know that they are approved/official

 I know that they are non-approved/non-official

 I don’t know

d. Did you get the receipt?

 Yes and what I paid matched with the receipt

 Yes, but I paid more than the receipt

 No, I didn’t get any receipt

23. Was there information on display or provided in another way about what people should pay for?

• Yes

• No

• Not sure/don’t know

24. Was there information on display or provided in another way about what is available for free?

• Yes

• No

• Not sure/don’t know

25. Were you surprised about having to pay?

• Yes

• No

• Not sure/don’t know

26. Did you know whether any of these payments/contributions were requested by a health worker for themselves rather than for the benefit of the patients/operation of the facilities (e.g. to improve supplies and conditions)?

• Yes. Go to Q26a

• No, all payments were for the patients/facilities

• Not sure/don’t know

26a. If yes, which kind of payment?

 Consultation

 Drugs

 Consumables/supplies

 Diagnostic test

 Administrative charge

 Jumping the queue

 Made any payment but not sure what it was for

27. Thinking of the overall expenditure, what impact did it have on your household finance?

• Severe impact (couldn’t meet the other basic need)

• Moderate (significant but still could fulfil other need / reduced by a moderate amount)

• Mild (just reduced by small amount)

• None

28. How did you get the money to pay for your visit?

• From my own income

• Helped by close family member/ member of the immediate household

• Helped by extended family

• Helped by friends

• Helped by a church/missionary/mosque

• Helped by a philanthropist

• Helped by an influential figure (including politician or godfather/mother)

• Helped by a health worker in the facility

• Paid by health insurance

• Took a loan (specify from whom)

• Unable to pay or contribute in kind

• Other way ___________________specify

• I didn’t have to pay for anything

• Not sure/DK

Favour to access health care

29. Do you or someone from your household help or provide favours to the health workers working in the facility you visited on the last occasion?

Probing for INTERVIEWER: do they usually or ever help in this way?

• Yes ________________specify

• No, skip to Q31

• Not sure/don’t know

30. What service/s or favour/s have you provided? (two answers)

• Running an errand

• Provision of labour or services

• Giving food items

• Helping them solve a problem (e.g. getting them in education or getting a job)

• Other___________________specify

• Not sure/DK

31. During the visits related to the latest occasion of visiting public healthcare facility in the last 6 months, if you or someone from your household made unapproved or an ‘extra’ payment, gave a gift, or did a favour for a staff member at the facility, when was this usually done? (One answer)

• Before the service was delivered

• After the service was delivered

• At the same time that the service was delivered

• Partly before and partly after the service was delivered

• Nothing was paid but payment is expected to happen in the future

• Not sure/DK

32. During any of the visits from the latest occasion in the last 6 months, has any service/medication been refused or delayed due to some of the following reasons: (tick all applies)

• Was refused/delayed, because I couldn’t pay

• Was refused/delayed, because I didn’t have connections

• Was refused/delayed for other reason/s ________________________________(specify)

• Was refused/delayed, and I am not sure for what reason, but suspect that it was because I could not pay or use connections

• There was not such case

• Not sure/DK

33. In your opinion, was the received health-related service worth the extra payment or gift?

• Yes

• No

• Not sure/don’t know

34. Have you or someone from your household refused to pay a bribe, give a gift, pay extra money (but not an official fee) or do a favour for a healthcare worker – in last 6 month?

• Yes

• No. Go to Q36

• Not sure/don’t know

35. If yes, on that occasion, did you or someone from your household still received a treatment or a service without paying a bribe, giving a gift, paying extra money (but not an official fee) or do a favour for a healthcare worker?

• Yes

• No

• Not sure/don’t know

The use of connections

36. For the last episode (6 month) we are discussing, did you have to use connections to receive care? Choose only one

• Paid unapproved/extra payment (to staff) but no connections were used

• Paid unapproved/extra payment (to staff) and I/we also used connections to secure treatment

• No payment but I/we used connections to secure treatment

• No payment and no connections were used

• Not sure/don’t know

37. If yes, what kind of connections were these:

• Householder members

• Close family

• Extended family (include the spouse’s family)

• Close friends

• People living in your community

• Community organisation

• Powerful people living in your community

• Politicians

• People in government (e.g. district, LGA)

• Other_________________specify

• Not sure/DK

Absenteeism/ Human Resource issues

38. Were the health workers present at the facility when you visited (the same occasion of visiting a facility discussed above/ last 6 months) or they were absent? (choose one)

• Yes, always there

• They were mostly at work

• They were mostly absent

• They are never there

• Not sure/don’t know

39. Were the health workers on time and working until the end of their shift when you visited (the same occasion of visiting a facility discussed above/ last 6 months) or they tended to be late/leaving early? (choose one)

• Yes, always on time

• They were mostly on time

• They were often arriving early

• They were often leaving early

• They were almost always late/leaving early

• Not sure/ don’t know

40. For the same occasion that we discussed (last 6 months), have you or someone from your household been in the situation when you or someone from your household had to wait and still did not receive treatment or the service you needed?

• Yes

• No

• Not sure/ don’t know

41. For that occasion, have you experienced a situation when you or your family member did not get treatment or the service you needed because a doctor or a nurse in a public healthcare facility was absent?

• Yes

• No

• Not sure/ don’t know

42. For that occasion, how often did you or your family member not get treatment because a doctor or a nurse in a public healthcare facility was absent?

• Always

• Often

• Several times

• Once or twice

• Never/Not sure/ don’t know

43. Have you experienced or observed cases where staff were at work but for no justifiable/explained reasons not attending to the patients, or ignoring them?

• Yes, frequently

• Sometimes

• Never there

• Not sure/DK

44. During the same occasion, did you suspect that there were staff member(s) who were not sufficiently qualified or experienced?

• Always

• Often

• Several times

• Once or twice

• Never

• Not sure/DK

Overall assessment of episode

45. On a scale of 1-5, how disruptive were any of these for your own (or the household members treatment) treatment or obtaining the other services?

a. Unofficial/ unapproved payments

Didn’t experience it 1 (not too disruptive) 2 3 4 5 (very disruptive)

b. Absenteeism

Didn’t experience it 1 (not too disruptive) 2 3 4 5 (very disruptive)

c. Referral of patients from public to private facilities

Didn’t experience it 1 (not too disruptive) 2 3 4 5 (very disruptive)

46. On the whole, did you feel that you received good service when seeking care for your last illness episode?

• Yes, very good

• Mostly adequate

• Mostly inadequate

• Poor

• Not sure/DK

II Vignettes

We will now tell you about a few situations which may occur in real life and we will ask you a few questions about these.

Interviewer: Ask respondents to think about someone like them

Scenario 1

[NIGERIA]

A friend of yours who lives nearby goes to deliver her baby at a health centre. They are asked for 3 boxes of gloves; 3 bottles of Dettol and 3 packets of cotton wool by the nurse caring for them. They know that they will only need one box of gloves, 1 bottle of Dettol and one packet of cotton wool for their care and that the nurse will sell the rest of these commodities.

47. Is this scenario common around here? Yes/No

48. What would you advise your friend to do? Choose one option only (main course of action)

a. My friend should leave the facility and give birth at home (helped by someone or traditional birth attendant)

b. My friend should report to the WDC

c. My friend should bargain with the nurse who is asking for the commodities and try to get her/him to reduce the demands

d. My friend should provide the commodities with the money they have

e. My friend should go to the Igwe/traditional leader to make a complaint.

f. My friend should go to the officer in charge (OIC) and make a complaint

g. My friend should complain to the head of department (HOD) or any powerful person in authority position away from the health facility

h. My friend should go to a locally powerful businessman and ask their help

i. My friend should go to another facility

j. Other _______ (specify)

49. Why do you make that choice? Choose one option only

a. There is no other choice

b. It is the most effective thing to do

c. It is the right thing to do (it’s what socially accepted)

d. Other ______ (specify)

Scenario 2

Your child is ill and you go to the health facility. When you arrive there is a very long queue and the nurses have come late to work. Someone in the queue told you that if you pay you can see a health worker more quickly.

50. Is this scenario common around here? Yes/No

51. What would you do? Choose one option only (main course of action)

a. You leave the facility and get self-treatment

b. You bargain to the nurse and explain how ill your child is and ask to be seen quickly without paying any extra money to jump the queue

c. You talk to the nurse and offer her some money

d. You go to see the Igwe/traditional leader and ask for help

e. You see the officer in charge (OIC) and ask for help

f. You complain to the head of department (HOD) or any powerful person in authority position away from the health facility

g. You see a locally powerful businessman and ask for help

h. Other ___________(specify)

52. Why do you make that choice? Choose one option only

a. There is no other choice

b. It is the most effective thing to do

c. It is the right thing to do (it’s what socially accepted)

d. Other ______ (specify)

Scenario 3

Your neighbour arrives at the health facility with a fever. A nurse performs a rapid test, and the nurse confirms that your neighbour has malaria. The nurse says that there are no free medicines available and that your neighbour will have to purchase XX for XX (a lot of them) and pay him/her directly (either by cash or a bank transfer to his/her personal bank account). You negotiate with the cleaner or porter to help you get the medicines.

53. Is this scenario common around here? Yes/No

54. What would you advise your neighbour to do? Choose one option only (main course of action)

a. My neighbour should leave the facility and get self-treatment

b. My neighbour should bargain to the nurse and try to reduce the demands

c. My neighbour should purchase what the nurse demands

d. My neighbour should go to see the Igwe/traditional leader and ask for help

e. My neighbour should see the officer in charge (OIC) and ask for help

f. My neighbour should complain to the head of department (HOD) or any powerful person in authority position away from the health facility

g. My neighbour should see a locally powerful businessman and ask for help

h. Other ___________(specify)

55. Why do you make that choice? Choose one option only

a. There is no other choice

b. It is the most effective thing to do

c. It is the right thing to do (it’s what socially accepted)

d. Other ______ (specify)

III Perceptions (rule-breaking/corruption and trust in institutions)

56. Did you feel it is right for health workers to request additional (non-approved) payment for:

a. Outpatient visits, such as: antenatal care, postnatal, family planning, malaria, HIV, tuberculosis, minor surgeries, wound dressing, or other yes/to some extent/no

b. Childbirth yes/to some extent/no

c. In-patient stay yes/to some extent/no

d. Drugs, consumables, supplies yes/to some extent/no

e. Routine immunization yes/to some extent/no

f. Diagnostic tests yes/to some extent/no

g. Certificates or other administrative charges yes/to some extent/no

h. Other __________________(specify)

57. If yes to some of the above, why? (choose two responses):

• Paying is the only way to get reasonable service

• Nothing is free

• Lack of insurance coverage

• Facilities lack resources

• Health workers are poorly paid

• The government doesn’t provide sufficient health budget

• It is required to get medicines

• It is a way of appreciating their good work

• Other __________________(specify)

58. Generally speaking, would you say that most people can be trusted or that you need to be very careful in dealing with people? (Code one answer):

• Most people can be trusted

• Not always, one needs to be careful

• Most people cannot be trusted

59. I‘d like to ask you how much you trust people from various groups. Could you tell me for each whether you trust people from this group completely, somewhat, not very much or not at all? (Read out and code one answer for each):

Trust completely Trust somewhat Do not trust very much Do not trust at all

A Your family 1 2 3 4

B Your neighbourhood 1 2 3 4

C People you know personally 1 2 3 4

D People you meet for the first time 1 2 3 4

E People of another religion 1 2 3 4

F People of another nationality/tribe 1 2 3 4

60. Do you have faith or believe that this actors have the power to contribute to reducing corruption?

Not powerful at all Completely powerful

1 2 3 4

A President of the country 1 2 3 4

B Government ministers 1 2 3 4

C State Governors 1 2 3 4

D House of assembly/house of representatives/senators 1 2 3 4

E Ward councilors (local government) 1 2 3 4

F Regulatory agencies 1 2 3 4

G Members of parliament 1 2 3 4

H Courts 1 2 3 4

I Army 1 2 3 4

J Police 1 2 3 4

K Church/ mosque/shrine 1 2 3 4

L Local government council

M Professional associations 1 2 3 4

N Traditional rulers (igwe or others in Nigeria) 1 2 3 4

O Political parties 1 2 3 4

P Newspapers actors/ owners 1 2 3 4

Q Television and radio actors/owners 1 2 3 4

R Social media personalities 1 2 3 4

S Nongovernmental organizations (NGOs)/ Civil Society organisations (CSOs) 1 2 3 4

T Ward Development Committees (WDCs) 1 2 3 4

U Trade/labour/ town unions 1 2 3 4

61. Do you have faith or believe that this actors are willing to contribute to reducing corruption?

Not powerful at all Completely powerful

1 2 3 4

A President of the country 1 2 3 4

B Government ministers 1 2 3 4

C State Governors 1 2 3 4

D House of assembly/house of representatives/senators 1 2 3 4

E Ward councilors (local government) 1 2 3 4

F Regulatory agencies 1 2 3 4

G Members of parliament 1 2 3 4

H Courts 1 2 3 4

I Army 1 2 3 4

J Police 1 2 3 4

K Church/ mosque/shrine 1 2 3 4

L Local government council

M Professional associations 1 2 3 4

N Traditional rulers (igwe or others in Nigeria) 1 2 3 4

O Political parties 1 2 3 4

P Newspapers actors/ owners 1 2 3 4

Q Television and radio actors/owners 1 2 3 4

R Social media personalities 1 2 3 4

S Nongovernmental organizations (NGOs)/ Civil Society organisations (CSOs) 1 2 3 4

T Ward Development Committees (WDCs) 1 2 3 4

U Trade/labour/ town unions 1 2 3 4

62. Do you have faith or believe that this actor have to tools and resources to contribute to reducing corruption?

No trust at all Complete trust

1 2 3 4

A President of the country 1 2 3 4

B Government ministers 1 2 3 4

C State Governors 1 2 3 4

D House of assembly/house of representatives/senators 1 2 3 4

E Ward councilors (local government) 1 2 3 4

F Regulatory agencies 1 2 3 4

G Members of parliament 1 2 3 4

H Courts 1 2 3 4

I Army 1 2 3 4

J Police 1 2 3 4

K Church/ mosque/shrine 1 2 3 4

L Local government council

M Professional associations 1 2 3 4

N Traditional rulers (igwe or others in Nigeria, mafumu in Malawi) 1 2 3 4

O Political parties 1 2 3 4

P Newspapers actors/ owners 1 2 3 4

Q Television and radio actors/owners 1 2 3 4

R Social media personalities 1 2 3 4

S Nongovernmental organizations (NGOs)/ Civil Society organisations (CSOs) 1 2 3 4

T Ward Development Committees (WDCs) 1 2 3 4

U Trade/labour/ town unions 1 2 3 4

63. Here are a few questions about people in your life who have the power to help you when seeking care outside immediate family [Interviewer: financial, material support, access to the right people]

Yes No Refused Don’t know

a. Is there anyone who you can really count on to help you out in a crisis/in your most difficult moment? 1 2 98 99

b. Is there anyone who you can borrow money from in a crisis?

64. To what extent do you agree or disagree with the following statements:

Strongly disagree Disagree Neither agree nor disagree Agree Strongly agree Don’t know Refused to answer

Most people in Nigeria/Malawi expect to pay something extra when they see a health care provider in a public health facility.

To receive good quality healthcare from a public facility in Nigeria, you must pay a non-approved or extra money, give a gift or do a favour to a health care worker.

If you do not pay a non-approved payment, give a gift or use a personal relationship, you will likely have to wait longer or receive services in a public health facility.

It is acceptable for health workers to receive non-approved payments.

Social capital

65. Now I am going to read off a list of voluntary organizations. For each organization, could you tell me whether you are an active member, an inactive member or not a member of that type of organization? (Read out and code one answer for each organization)

Active member Inactive member Don’t belong

A Religious organisation 1 2 3

B Political party at community level 1 2 3

C Political party at LGA/district level 1 2 3

D Professional association 1 2 3

E Ward Development Committee (Nigeria) 1 2 3

F Facility health committees 1 2 3

G Women’s group 1 2 3

H Other (specify….) 1 2 3

66. If you have a serious problem, who do you go to for help? (Multiple choice)

• Powerful family members

• Powerful close friends or friends with connections to powerful people

• Powerful people living in your community

• Powerful members of community organisations (from WDCs, Health Facility Committees)

• Powerful people at district or LGA level (e.g. Politicians, HoD)

• Godfather/ godmother

• Powerful people at national level (e.g. Politicians)

• Other_________________specify

• Not sure/don’t know

67. Have you ever asked for and/or received help to obtain treatment or another service from health facilities from the following people?

• Powerful family members

• Powerful close friends or friends with connections to powerful people

• Powerful people living in your community

• Powerful members of community organisations (from WDCs, Health Facility Committees)

• Powerful people at district or LGA level (e.g. Politicians, HoD)

• Godfather/ godmother

• Powerful people at national level (e.g. Politicians)

• Other_________________specify

• Not sure/don’t know

IV Complaints process

68. Have you ever complained about any of the following problems in the health system?

• Being asked to pay

• Health workers being late for work or leaving too early

• Health workers being absent from the facility

• Facilities lacking basic equipment

• Health workers engaging in economic activities within the premises

• Being asked for sexual favours to receive better treatment

• Other _________________ specify

• Not sure/ don’t know

• Never made any complaint. Skip to Q72

69. To whom have you ever complained to? (any time in the past, not just last episode)

• Managers – Head of Department

• Managers – Officer in charge

• Facility health committees

• Ward Development Committees

• Doctors/nurses in the facility

• Traditional rulers/leaders/ Igwe

• President general (community leaders)

• Local politicians

• Influential community member

• Godfathers/godmothers

• To any informal structures/ social and kinship networks

• To bishops/religious leaders

• Social media complaints

• Other _________________ specify

• Not sure/ don’t know

70. Was the problem resolved?

• Yes, the problem was resolved

• Some action taken but the problem was not fully resolved

• No, not resolved

• Not sure/ don’t know

71. Did you get feedback on how the complaint is resolved and what action was taken?

• Yes, to some extent, formally

• Yes, to some extent, informally

• No

• Not sure/ don’t know

72. Do you know someone who has complained to any of these people/institutions?

• Managers – Head of Department

• Managers – Officer in charge

• Facility health committees

• Ward Development Committees

• Doctors/nurses in the facility

• Traditional rulers/leaders/ Igwe

• President general (community leaders)

• Local politicians

• Influential community member

• Godfathers/godmothers

• To any informal structures/ social and kinship networks

• To bishops/religious leaders

• Social media complaints

• Other _________________ specify

• Not sure/ don’t know

73. What was the outcome?

• Yes, the problem was resolved

• Some action taken but the problem was not fully resolved

• No, not resolved

74. Are you aware of cases where staff at health facilities should have been sanctioned but were not, and why?

• Not aware of such cases

• Yes, the person had protection by politicians

• Yes, the person had protection by powerful community members

• Yes, the person had protection by godfathers

• Yes, the person had protection by health system managers/officials

• Not sure/ don’t know

75. What category of people tend to protect the health workers when they behave badly/in corrupt ways towards patients?

• Traditional rulers

• High ranking health official

• Federal/State level politician

• Local government official

• Local politician (e.g. ward councillor)

• Powerful influential person in the community

• Nobody can protect people

• Not sure/ don’t know

76. If you or your close relative face corrupt practices, what do you do?

• You approach health structures

• You approach local government structures

• You approach facility health committees

• You approach traditional ruler/, community or religious leaders

• You approach neighbour/relative or social networks

• Other ___________________ (specify)

• No such action usually/nothing can be done

• Not sure/ don’t know

77. What can be done to reduce any unapproved or unethical practices in health facilities you use (unapproved payment to staff, absenteeism, favouritism or others)?

• Make a complain

• Seek help from powerful individuals (formal organisations

• Seek help from powerful individuals (informal – godfathers, political connections, personal networks etc.)

• Change of political regime

• Other _____________________________(specify)

• Not much can be done currently

V Demographic and socio-economic information

All relevant boxes MUST be filled.

Interviewer read: This section is designed to help us learn about you and your household. Please take a few minutes to answer the following questions. Some questions can be answered by observing the respondent.

I would like to begin by asking a few questions about your household.

78. Household size: How many people live in this household, including yourself? [ ]

Interviewer note: remember the definition of a household: a person or group of related or unrelated persons who live together in the same dwelling unit(s), who acknowledge one adult male or female as the head of the household, who share the same housekeeping arrangements and who are considered a single unit.

79. Household composition (simple) How many household members belong to the following age groups?

0-5 years

6-12 years

13-19 years

20-44 years

45-65 years

more than 65 years

Total (should match to the response to Q64)

Note to interviewer: If the respondent answers section I about their own visits to facility or on behalf of a minor (<17 year old), a dependent, someone who is too old or too sick to answer, we collect the respondent’s information from here on. But if they answers section I on behalf of another adult that can make decision by themselves, please collect information from that adult.

80. Household role: What is your status in this household? [ ]

0 = female head of household; 1 = male head of household; 2 = wife; 3=husband; 4 = grandmother; 5=grandfather; 6 =other member representing the household

81. Education level. What was your highest completed education level? Choose one

Nigeria:

a. I didn’t go to a school Skip to Q83

b. Primary school

c. Junior secondary

d. Senior secondary

e. University

f. Polytechnics

g. Educational colleges

h. Other, specify:………….

Malawi:

a. I didn’t go to a school

b. Primary school

c. Secondary school

d. University

e. Other, specify:………….

82. Years of education. What was the total number of years that you spent schooling? [ ] years

83. Occupation. What occupation is your major source of income? [ ]

0 = Unemployed; 1 = Student/Learner; 2 = Housewife; 3 = Subsistence farming; 4 = Pensioner

5 = Petty trading; 6 = Artisanal work (welder, mason, labourer, etc) 7 = Government Worker;

8 = Employed in private sector; 9 = Owns a business; 10 = Self-employed professional; 11 = Others; 12 = Don’t know

84. Marital status: What is your current marital status? [ ]

0 = never married (single) 1 = living with spouse. 2 = widowed. 3 = divorced/separated.

85. Income Sufficiency: Which one of these phrases comes closest to your own feelings about your household’s income these days?

a. Living comfortably on current income

b. Getting by on current income

c. Finding it difficult on current income

d. Finding it very difficult on current income

86. Self-perceived financial situation. How would you describe the economic situation of your household this year compared to other people who live in your area?

1 = Very good 98 = Refusal

2 = Good 99 = Don't know

3 = Average

4 = Bad

5 = Very bad

87. Income sufficiency. How often in the past twelve months did your family have to reduce the use of the following:

Constantly Sometimes Never Do not use it

a. Basic foods 1 2 3 4

b. Essential clothes, shoes 1 2 3 4

c. Electricity 1 2 3 4

d. Transportation/fuel for car 1 2 3 4

e. Health care (or a services from a health facility) 1 2 3 4

f. Medicines and consumables 1 2 3 4

g. Home repairs 1 2 3 4

Household characteristics – This is about the household so the respondent can ask someone else to provide correct information

88. Asset ownership. Could you tell me if you or someone in your household owns these items and whether they are still functional? (If YES and functional), How many do you (he or she) own?

Ownership Yes/No Functional

Yes/No How many?

Electronics

Radio

Fridge

Television

Air conditioner

Computer/laptop

Mobile phone

Tablet

Transport

Bicycle

Motorcycle

Car

Lighting

Kerosene lamp

Generator

Rechargeable lamp

89. Does any member of this household have a bank account? Yes/No

90. Is this dwelling unit (house) owned, rented or provided by institutions / employer?

a. Owned/Family

b. Rented

c. Institutional

d. Other (specify)……………………………………………

91. What is the main material used for the roof?

Natural roofing

a. No roof

b. Grass thatch/palm leaf

Rudimentary roofing

c. Rustic mat

d. Palm/bamboo

e. Wood planks

f. Cardboard

Finished roofing

g. Metal/zinc

h. Wood

i. Calamine/cement fibre

j. Ceramic tiles

k. Cement

l. Roofing shingles

m. Other: specify:….

92. What is the main material used for the wall?

Natural walls

a. No walls

b. Cane/palm/trunks

c. Dirt

Rudimentary walls

d. Bamboo with mud

e. Stone with mud

f. Uncovered adobe

g. Plywood

h. Cardboard

i. Reused wood

Finished walls

j. Cement

k. Stone with lime/cement

l. Bricks

m. Cement blocks

n. Covered adobe

o. Wood planks/shingles

p. = Other (Specific) ………………………………………

93. What is the main material used for the floor?

Natural floor

a. Earth / sand

b. Dung

Rudimentary floor

c. Wood planks

d. Palm/bamboo

Finished floor

e. Broken bricks

f. Parquet/polished wood

g. Vinyl or asphalt strip

h. Ceramic/porcelain tiles

i. Cement

j. BRICKS

k. OTHER (specific) ……………………………….

94. What is the type of dwelling structure?

(Based on material used, select the type of dwelling)

a. = Permanent

b. = Semi-permanent

c. = traditional

95. What is the source of energy the household mainly uses for cooking?

a. Electricity

b. LPG

c. Natural Gas

d. Biogas

e. Kerosene

f. Coal/lignite

g. Charcoal

h. Wood

i. Straw/shrubs/grass

j. Agricultural crop

k. Animal dung

l. No food cooked in household

m. Other. Please specify:……

96. How many rooms in this household are used for sleeping? _____________(specify)

97. What is your household source of water?

a. Piped Private

b. Piped Public

c. Well

d. Surface water e.g. stream, river, etc.

e. Water tanker supplies water in home tank

f. Rainwater

g. Other. Please, specify: _________________________________________

98. What kind of toilet facility do you have in your home? 1 = yes 0 = no

Flush or pour flush toilet

a. Flush to piped sewer system

b. Flush to septic tank

c. Flush to pit latrine

d. Flush to somewhere else

e. Flush, don’t know where

Pit Latrine

f. Ventilated improved pit latrine

g. Pit latrine with slab

h. Pit latrine without slab/open pit

i. Composting toilet

j. Bucket toilet

k. Hanging toilet/hanging latrine

l. No facility/Bush/Field

m. Other, please specify:…………

99. Where is this toilet facility located?

a. In own dwelling

b. In own yard/plot

c. Elsewhere

INTERVIEWER: Please record:

What time did the interview end: __________________________?

THANK YOU
